# Characterization of Circulating Natural Killer Cells in Neotropical Primates

**DOI:** 10.1371/journal.pone.0078793

**Published:** 2013-11-11

**Authors:** Angela Carville, Tristan I. Evans, R. Keith Reeves

**Affiliations:** Division of Immunology, New England Primate Research Center, Harvard Medical School, Southborough Campus, Southborough, Massachusetts, United States of America; Tulane University, United States of America

## Abstract

Despite extensive use of nonhuman primates as models for infectious diseases and reproductive biology, imprecise phenotypic and functional definitions exist for natural killer (NK) cells. This deficit is particularly significant in the burgeoning use of small, less expensive New World primate species. Using polychromatic flow cytometry, we identified peripheral blood NK cells as CD3-negative and expressing a cluster of cell surface molecules characteristic of NK cells (i.e., NKG2A, NKp46, NKp30) in three New World primate species – common marmosets, cotton-top tamarins, and squirrel monkeys. We then assessed subset distribution using the classical NK markers, CD56 and CD16. In all species, similar to Old World primates, only a minor subset of NK cells was CD56+, and the dominant subset was CD56–CD16+. Interestingly, CD56+ NK cells were primarily cytokine-secreting cells, whereas CD56–CD16+ NK cells expressed significantly greater levels of intracellular perforin, suggesting these cells might have greater potential for cytotoxicity. New World primate species, like Old World primates, also had a minor CD56–CD16– NK cell subset that has no obvious counterpart in humans. Herein we present phenotypic profiles of New World primate NK cell subpopulations that are generally analogous to those found in humans. This conservation among species should support the further use of these species for biomedical research.

## Introduction

In primates, there are two major arms of the immune system: a) antigen-specific adaptive immunity and b) antigen-independent innate immunity. Innate immune responses limit the spread and subsequent tissue destruction of bacterial and viral infections and nascent neoplasms before the onset of adaptive immunity, but also contribute to the shaping of adaptive immune responses by cellular editing and cytokine secretion. The primary effector cells of the innate immune system are natural killer (NK) cells, which can have both cytotoxic and cytokine-based regulatory functions. Indeed critical roles for NK cells in defense against a number of viral infections including influenza, CMV, VZV, and HSV [1]–[6] have been documented, but NK cells also play important modulatory roles such as in pregnancy [7]–[9]. NK cells have evolved multiples mechanisms for the recognition of aberrant cells, the primary basis of which rests on a two-signal discrimination of “self” versus “non-self”: a positive signal initiating lysis and an inhibitory signal that is necessary to prevent lysis. The first signal is an interaction with cell-surface MHC, which would be expressed on healthy cells, but lost on many virus-infected or stressed cells. A second signal can involve so-called natural cytotoxicity receptors (NCRs) including NKG2a, NKp30, NKp44, and NKp46, which can be inhibitory or activating [10], [11]. In recent years attention has been more focused on MHC interactions with killer immunoglobulin-like receptors (KIRs), a polygenic family of NK cell surface receptors that appear to mediate NK cell activation and cytolysis in humans and nonhuman primate species, but are absent in other mammals [11]–[17]. NK cell expression of the low affinity FcγR, CD16, which binds antibodies coated on targeted cells, can also regulate antibody-dependent cell-mediated cytotoxicity. Through this complex discrimination, NK cells maintain balance of tolerance and cytotoxicity.

In humans, two primary subsets of NK cells are found, cytolytic CD56^dim^CD16^+^ and cytokine-secreting CD56^bright^CD16^−^ subsets, of which the CD56^dim^CD16^+^ subset predominates in blood. Efforts to identify comparable populations of NK cells in nonhuman primate models were complicated by incomplete definitions, but we have more recently determined a definition of CD3^–^CD8αα^+^CD20^−/dim^NKG2A^+^ is one of the most effective inclusive definitions for Old World monkeys such as rhesus and pig-tailed macaques [18]–[20]. Other groups have found this definition to be similarly effective for sooty mangabeys [21]. Like humans, Old World monkey NK cell subpopulations include cytolytic CD16^+^CD56^−/dim^ and cytokine-secreting CD16^−/dim^CD56^hi^ cells, but also multifunctional CD16^–^CD56^–^ NK cells which have no obvious counterpart in humans [20].

To date, evaluations of NK cell populations in New World (neotropical) primate species have been limited, and often have used nonspecific NK cell markers [22]–[26]. Such limitations have been imposed, at least partially, by limited numbers of known cross-reactive antibodies in these species. However, in recent years a wide range of neotropical primate disease models have been developed including those for EBV, KSV, HCV, lymphoma, neurodegenerative disorders, and autoimmune diseases [25]–[32]. With hundreds of millions of persons affected by these diseases worldwide, a need for better tools to study immune responses in these models has arisen. Therefore, in this study we sought to comprehensively characterize the phenotypic and functional biology of NK cells in neotropical primate model species use comprehensive polychromatic flow cytometry (PFC) panels.

## Methods

### Ethics Statement

Animals were housed at the New England Primate Research Center (NEPRC) and were maintained in accordance with the guidelines of the local institutional animal care and use committee and the Department of Health and Human Services (DHHS) Guide for the Care and Use of Laboratory Animals. All animals were socially housed and enrolled in the NEPRC environmental enrichment program designed to provide mental and sensory stimulation and promote development of behavioral and logical skills using varied stimuli (i.e., foraging devices). Samples obtained for analysis in this study were collected during routine physical examinations, performed quarterly at NEPRC. Animals were not sedated specifically for biomedical manipulation, but rather blood draws were timed to coincide with routine health-checks thereby eliminating any unnecessary pain or distress. Thus no blood was drawn specifically for research purposes, but excess sample that would have otherwise been discarded was allocated for these analyses. Blood draws consisted of no more than 1% of body weight and not more than 3 ml, and were performed by Angela Carville, B.V.M.S., a clinical veterinarian with over 20 years experience specific to working with nonhuman primates, including neotropical primates. Post-blood draw analgesics were administered at the discretion of the veterinarian.

### Animals

Neotropical primates used in this study included common marmosets (*Callithrix jacchus*, n = 14), cotton-top tamarins (*Saguinus oedipus*, n = 8); and squirrel monkeys (*Saimiri sciureus*, n = 6). Animals were fed a commercial new world nonhuman primate diet (New World Primate Chow 8791, Harlan Teklad, Indianapolis, IN), which was supplemented with fruits, vegetables, eggs and nuts. Water was available ad libitum.

### Polychromatic Flow Cytometry

Flow cytometry staining of mononuclear cells was carried out for cell-surface and intracellular molecules using standard protocols [20]. LIVE/DEAD Aqua dye (Invitrogen) and isotype-matched controls and/or fluorescence-minus-one (FMO) controls [33] were included in all assays. Acquisitions were made on an LSR II (BD Biosciences) and analyzed using FlowJo software (Tree Star Inc., Ashland, OR).

### NK Stimulation Assay

To analyze functional NK cell responses ex vivo we used a modification of an assay previously developed in our laboratory for Old World primates [20], [34]. Briefly, 1×10^6^ NK-enriched cells were resuspended in RPMI 1640 (Sigma-Aldrich) containing 10% FBS and stimulated at an E:T ratio of 1∶1 with 721.221 cells, or with PMA (50 ng/ml) and ionomycin (1 µg/ml), or with medium alone. Golgiplug (brefeldin A) and Golgistop (monensin) were added at final concentrations of 6 µg/ml, then all samples were cultured for 12 hours at 37°C in 5% CO_2_. After culture, samples were surface-stained using markers to delineate NK cells (CD3, NKp46, CD16, CD56). Cells were then permeabilized using Caltag Fix & Perm and intracellular cytokine staining was performed for IFN-γ (APC conjugate, clone B27, Invitrogen) and TNF-α (Alexa 700 conjugate, clone Mab11, Pharmingen).

### Statistical Analyses

All statistical and graphical analyses were done using GraphPad Prism (version 6.0a) software (GraphPad Software, Inc., La Jolla, CA). Nonparametric Mann-Whitney U and Wilcoxon matched pairs tests were used where indicated and *P*<0.05 were assumed to be significant in all analyses.

## Results

### Identification and Quantification of Natural Killer Cells in Neotropical Primates

**Figure 1:**
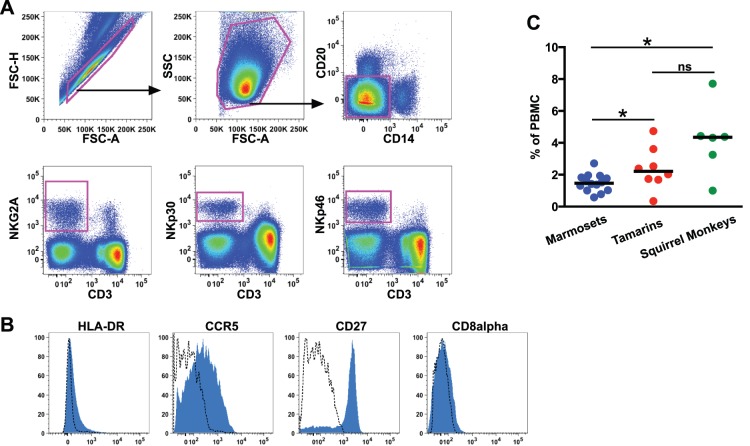
Peripheral blood NK cells in neotropical primate species. (**A**) Representative gating strategy identifying circulating NK cells. (**B**) Representative histograms of secondary molecule expression on NK cells as gated in A. (**C**) Cross-sectional comparisons of NKp46+ NK cell frequencies among total mononuclear cells in different neotropical primate species. Bars represent medians. Mann-Whitney *U* tests were used for interspecies comparisons; *, *P*<0.05; ns, not significant.

Since little research had previously been performed on NK cells in neotropical primate species we initially used gating strategies from other primate models as a guide for identification. We tested cross-reactivity of a battery of monoclonal antibodies often used in other nonhuman primates in three neotropical species – common marmosets, cotton-top tamarins, and squirrel monkeys. Using up to 8-color flow cytometry we identified a number of cross-reactive antibodies to NK cell and lineage markers that could be used specifically in these species (Figure S1). We were able to identify a population of large granular lymphocytes that were non-B (CD20–) non-monocytic (CD14–) non-T cells (CD3–) in all species. To further confirm these cells as NK cells we used antibodies to three NK-cell specific markers, NKG2A, NKp30, and NKp46. These three antibodies are among few available antibodies to NK cell markers that have any cross-reactivity in other nonhuman primate species [20], [21], [34], [35]. Although in marmosets the three primary NK cell antibodies were highly cross-reactive, there was variable cross-reactivity for both anti-NKG2A and anti-NKp30 in tamarins and squirrel monkeys (Table 1). This could be due to some allelic variation in the epitope targeted by these antibodies (unpublished observations). However, anti-NKp46 exhibited bright staining in all species, thereby allowing us to use an NK cell inclusive definition of large lymphocytes CD3–CD14–CD20–NKp46^+^ (Figure 1A). Furthermore, in those animals where the antibodies to NKp30 and NKG2a were cross-reactive, both molecules were co-expressed with NKp46, suggesting these molecules similarly identify NK cells in each of the species (Figure S2). Although this may not be an all-inclusive definition for NK cells, at present it is the most comprehensive proposed working definition for these species. In addition to NK cell-specific markers we also examined a number of cell-surface markers that are associated with NK cell development, trafficking and activation (Figure 1B). NK cells from the species examined expressed little HLA-DR, but had variable levels of CCR5 and high cell surface expression of CD27, consistent with other nonhuman primate and human NK cells. Interestingly, CD8α was negative to dim on NK cells from all neotropical species (Figure 1B, Figure S3); an unexpected finding since CD8α is expressed at high levels on NK cells in Old World primates and delineates a subpopulation of NK cells in humans [20], [21], [34], [36], [37].

**Table 1:** Variable cross-reactivity of NK cell-specific antibodies among species.

	Marmosets	Tamarins	Squirrel Monkeys
**anti-NKG2A**	8/8* (100%)	2/8 (25%)	0/6 (0%)
**anti-NKp30**	14/14 (100%)	8/8 (100%)	4/6 (66.6%)
**anti-NKp46**	14/14 (100%)	8/8 (100%)	6/6 (100%)

* , number of animals evaluated.

Using the CD3^–^CD14^–^CD20^–^NKp46^+^ definition for NK cells we could then assess the frequency of NK cells in the circulation of neotropical species (Figure 1C). In general NK cells were most uncommon in marmosets with a median frequency of 1.5% of peripheral lymphocytes, compared to tamarins and squirrel monkeys, with median frequencies of 2.2% and 4.5%, respectively. Interestingly, the frequency of NK cells in each of these species was markedly rare compared to humans and other primate species that range from 5% to 40% [18], [20], [21], [34]–[36], [38]. However, whether this might be reflective of technical issues in identifying NK cells in these species or a true biologic difference is unclear.

### Disparate Subset Distribution of Circulating Natural Killer Cells in Primate Species

**Figure 2:**
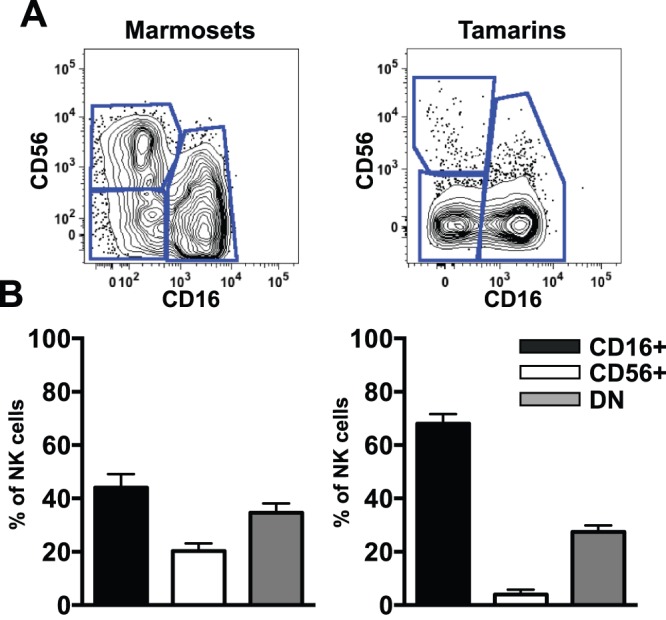
Disparate frequencies of NK cell subpopulations among neotropical primate species. (**A**) Representative flow cytometry plots displaying NK cell subpopulations based on CD56 and CD16 expression gated on NKp46+ NK cells as shown in Figure 1. (**B**) Graphic comparisons of NK cell subpopulations among common marmosets and cotton-top tamarins (lower panels). DN, double-negative.

In both humans and Old World primate species, such as rhesus macaques, CD56 and CD16 delineate subpopulations of cytokine-secreting and cytolytic natural killer cells, respectively. Similarly we found that CD56 and CD16 could be used to subdivide circulating NK cells into three subpopulations in marmosets and tamarins. – CD56^+^CD16^–^ (CD56^+^), CD56^–^CD16^+^ (CD16^+^), and CD56^–^CD16^–^ (DN) (Figure 2). This pattern of expression was more similar to that observed in Old World nonhuman primates where CD56 delineates only a small subset of NK cells compared to humans and chimpanzees where CD56 is expressed on virtually all NK cells [20], [34], [39]. However, the frequency of NK cell subpopulations among both neotropical species was somewhat disparate where, like in humans and in other primates, the CD16^+^ subpopulation was dominant in blood, followed in frequency by the DN subpopulation. Interestingly, in marmosets the CD56^+^ population was disproportionately large, representing a median of 20% of marmoset NK cells compared to less than 5% in tamarins. This unusual finding was, however, biologic and not due to cross-reactivity issues given the bright staining of CD56 on other tamarin non-NK lymphocytes (Figure S4).

### Functional Profiles of Natural Killer Cells in Neotropical Primates

**Figure 3:**
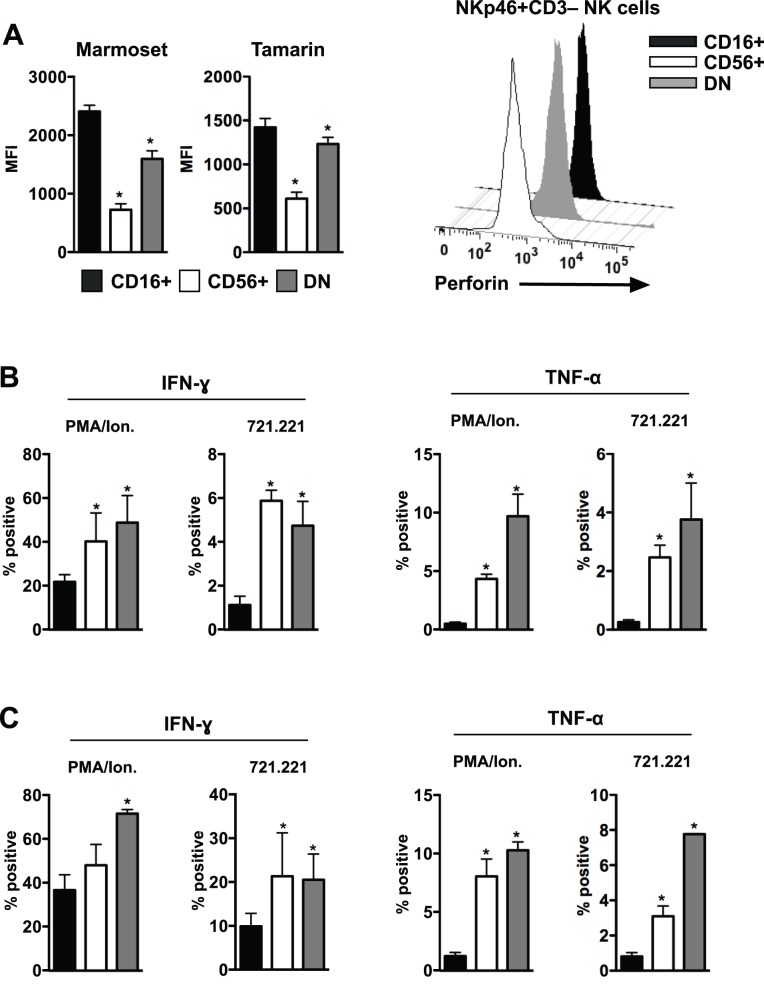
Functional profiles of marmoset and tamarin NK cells. (**A**) MFIs and example histograms of intracellular perforin expression in different NK cell subpopulations *ex vivo*. Percentages of peripheral blood NK cells from common marmosets (**B**) or cotton-top tamarins (**C**) positive for intracellular IFN-γ and TNF-α following PMA/ionomycin or 721.221 stimulation. Bars represent means ± SEM of 6 animals per group. Wilcoxon matched pairs tests were used for CD16+ NK cells versus CD56+ or DN NK cells; *, *P*<0.05. Only statistically significant differences are indicated.

In humans and Old World monkeys CD16^+^ NK cells are generally more cytotoxic while CD56^+^ NK cells are more cytokine-secreting cells. Unfortunately antibodies tested to two surrogate indicators of cytotoxicity, granzyme B and CD107a, were found to not cross-react in neotropical primates (Figure S1 and unpublished observations). However, an anti-perforin antibody had very strong cross-reactivity in all species for intracellular staining (Figure 3A) and we used this as a surrogate marker for cytotoxic potential of various NK cell subpopulations. Intracellular perforin expression was ∼2.5-fold greater in CD16^+^ compared to CD56^+^ NK cells, with expression in DN NK cells measuring intermediately between these two populations. Interestingly, perforin expression was generally greater (as measured by MFI) in marmosets compared to tamarins. Whether this is a biologic difference, or, alternatively, a relative cross-reactivity issue between the two species, is not clear.

To discriminate cytokine production profiles of NK cell subpopulations in New World primates, we next evaluated cells from both marmosets and tamarins in an *ex vivo* intracellular cytokine staining assay adapted from previous assays used in Old World primates and humans [19], [20], [34]. We evaluated production of IFN-γ and TNF-α in response to both mitogen (PMA/ionomycin) and the classical NK cell stimulus, MHC-devoid 721.221 cells. NK cells from marmosets and tamarins (Figure 3B and 3C) produced both cytokines at similar frequencies, although, not surprisingly, cytokine secretion was generally higher in response to PMA/ionomycin compared to 721.221 cells. IFN-γ was produced at similar levels by CD56^+^ and DN NK cells, whereas CD16^+^ NK cells generally produced much less. Similarly, TNF-α was produced predominantly by DN NK cells and CD56^+^ NK cells, but very little by CD16^+^ NK cells. This delineation of CD56^+^ NK cells as primarily cytokine-secreting cells is similar to what has been observed in other primate species, but additional experiments will be required to determine if the high level of perforin expression in CD16^+^ NK cells is actually indicative of increased cytolytic function [20], [34], [36], [40].

## Discussion

Herein we present for the first time an inclusive phenotypic definition of NK cells in neotropical primates using modern PFC techniques. Furthermore, our data suggest that the functionality of various NK cell subpopulations is conserved in New and Old World primates as well as humans. Given the increasing interest in use of neotropical primates in biomedical research, these data are highly relevant for establishing the validity and utility of such models for infectious disease and immunologic models. In addition, these data will provide a significant reference for other scientists on the cross-reactivity of a number of human antibodies in neotropical primate studies.

Using PFC we validated unequivocally the identity of neotropical primate NK cells and their subsets by demonstrating expression of NKG2A and NKp30 expression in addition to the more definitive marker NKp46. Others and we have previously determined that these markers are also inclusive for NK cells for rhesus and pig-tailed macaques and sooty mangabeys [18], [20], [21]. Like in other species, neotropical primate NK cells lacked expression of CD14, CD20, and HLA-DR, but could be divided into subpopulations by CD56 and CD16 expression. Although the paucity of CD56 expression on neotropical primate NK cells is more similar to Old World monkeys than the defining expression found in humans and apes. Perhaps most convincingly to confirm these as true NK cells; each of the subsets (CD56+, CD16+, DN) was responsive to the classical NK cell stimulus, MHC-devoid 721.221 cells. The DN NK cell subset is of particular interest since functionally and phenotypically it appears to be conserved among New and Old World monkeys, but does not have an obvious human counterpart and further studies will be required to delineate the ontogeny. However, neotropical primate NK cells expressed little to no CD8α. This is somewhat of an unexpected finding given that CD8α is often used as an NK cell-delineating marker in Old World primates and chimpanzees [20], [34], [41]. Although these data will need to be confirmed with subsequent studies, it is tempting to speculate that this could have an impact on NK cell functionality.

Although there was significant variability in the frequencies, we found that in each of the three neotropical primate species NK cells generally represented less than 5% (medians) of circulating mononuclear cells. This is in contrast to Old World monkeys and humans where NK cell frequencies are also highly variable, but generally much greater in number, up to 40% of lymphocytes [20], [34], [39]. There could be several explanations for this disparate pattern: 1) Our working definition for NK cells is incomplete and that we are not identifying the full complement of NK cells; or 2) NK cells are truly biologically more rare in neotropical primate species. The former is a caveat that we must accept as a possibility from our study. The second explanation, if true, could be of significant interest and have several plausible mechanisms. In general NK cells have two primary mechanisms for discrimination of self versus non-self, either NKG2 molecules or KIR binding to MHC. Unlike higher primates that express NKG2a, NKG2c, and NKG2e, marmosets only express one ancestral NKG2 molecule [42]. Furthermore, marmosets express a simplified repertoire of KIR molecules comprised of a single novel lineage related to human KIR3DL which co-evolved with the limited MHC also found in these species [17]. These data could suggest New World monkeys have a contracted NK cell repertoire in general. The simplified NK cell arm of neotropical primates might suggest they are more susceptible to certain viruses or tumors, and could also explain in part the unique immunologic tolerance in these species [30], [43]–[45]. Although the underlying reasons for modest differences between neotropical primate NK cells and those found in Old World monkeys and humans are not entirely clear, these data do suggest the basic functional “divisions of labor” are conserved and support the use of these species for future biomedical studies.

## Supporting Information

Figure S1 **Cross-reactivity of antibodies to neotropical primate antigens.** List of antibodies tested for cross-reactivity in neotropical primate species. Green (+) indicates consistent positive staining in the species whereas red (–) indicates lack of positive staining. Gray (+/−) indicates inconsistent cross-reactivity.
(EPS)

Figure S2 **Correlative expression of NK cell markers.** Percentages of NKp30^+^ (**A**) and NKG2A^+^ (**B**) NK cells in neotropical primate species correlate with percentages of NKp46^+^ NK cells, suggesting these markers identify equivalent cell types. Spearman’s correlation test (*r*), *P* values <0.05 are considered significant.
(EPS)

Figure S3 **Cross-reactive staining of anti-CD8α (B9_11) on neotropical primate lymphocytes.**
(EPS)

Figure S4 **Cross-reactive staining of anti-CD56 on tamarin lymphocytes.**
(EPS)
